# Integrative and Conjugative Elements and Prophage DNA as Carriers of Resistance Genes in *Erysipelothrix rhusiopathiae* Strains from Domestic Geese in Poland

**DOI:** 10.3390/ijms25094638

**Published:** 2024-04-24

**Authors:** Marta Dec, Aldert Zomer, John Webster, Tomasz Nowak, Dagmara Stępień-Pyśniak, Renata Urban-Chmiel

**Affiliations:** 1 Department of Veterinary Prevention and Avian Diseases, University of Life Sciences in Lublin, 20-033 Lublin, Poland; dagmara.stepien@up.lublin.pl (D.S.-P.); renata.urban@up.lublin.pl (R.U.-C.); 2 Division of Infectious Diseases and Immunology, Faculty of Veterinaty Medicine, Utrecht University, 3584 CL Utrecht, The Netherlands; a.l.zomer@uu.nl; 3 WOAH Reference Laboratory for Campylobacteriosis, WHO Collaborating Centre for Reference and Research on Campylobacter and Antimicrobial Resistance from a One Health Perspective, 3584 CL Utrecht, The Netherlands; 4 NSW Department of Primary Industries, Elizabeth Macarthur Agricultural Institute, PMB 4008, Narellan, NSW 2570, Australia; john.webster@dpi.nsw.gov.au; 5 Diagnostic Veterinary Laboratory “Vet-Lab Brudzew Dr. Piotr Kwieciński”, 62-720 Brudzew, Poland; tomasz@labbrudzew.pl

**Keywords:** *Erysipelothrix rhusiopathiae*, resistance genes, WGS, integrative and conjugative elements, prophage

## Abstract

Goose erysipelas is a serious problem in waterfowl breeding in Poland. However, knowledge of the characteristics of *Erysipelothrix rhusiopathiae* strains causing this disease is limited. In this study, the antimicrobial susceptibility and serotypes of four *E. rhusiopathiae* strains from domestic geese were determined, and their whole-genome sequences (WGSs) were analyzed to detect resistance genes, integrative and conjugative elements (ICEs), and prophage DNA. Sequence type and the presence of resistance genes and transposons were compared with 363 publicly available *E. rhusiopathiae* strains, as well as 13 strains of other *Erysipelothrix* species. Four strains tested represented serotypes 2 and 5 and the MLST groups ST 4, 32, 242, and 243. Their assembled circular genomes ranged from 1.8 to 1.9 kb with a GC content of 36–37%; a small plasmid was detected in strain 1023. Strains 1023 and 267 were multidrug-resistant. The resistance genes detected in the genome of strain 1023 were *erm47*, *tetM*, and *lsaE-lnuB-ant(6)-Ia-*s*pw* cluster, while strain 267 contained the *tetM* and *ermB* genes. Mutations in the *gyrA* gene were detected in both strains. The *tetM* gene was embedded in a Tn*916*-like transposon, which in strain 1023, together with the other resistance genes, was located on a large integrative and conjugative-like element of 130 kb designated as ICEEr1023. A minor integrative element of 74 kb was identified in strain 1012 (ICEEr1012). This work contributes to knowledge about the characteristics of *E. rhusiopathiae* bacteria and, for the first time, reveals the occurrence of *erm47* and *ermB* resistance genes in strains of this species. Phage infection appears to be responsible for the introduction of the *ermB* gene into the genome of strain 267, while ICEs most likely play a key role in the spread of the other resistance genes identified in *E. rhusiopathiae.*

## 1. Introduction

*Erysipelothrix rhusiopathiae* is a Gram-positive non-motile narrow rod-shaped bacterium with high growth requirements. It belongs to the genus *Erysipelothrix*, family *Erysipelotrichaceae*, order Erysipelotrichales, class Erysipelotrichia, phylum Bacillota (Firmicutes) [1,2]. While pigs are considered the main reservoir of *E. rhusiopathiae*, this bacterium can also cause infections in other vertebrates, including ruminants, rodents, birds, fish, cetaceans, and humans [3,4]. The pathogen may be excreted by infected animals in feces, urine, saliva, and nasal secretions, which may contribute to its spread in the environment by contaminating soil and water [5,6]. In pigs, the disease most often takes a subacute form with characteristic red skin lesions. Chronic erysipelas is generally associated with arthritis, lameness, and endocarditis. Less commonly, an acute form of the disease has occurred in pig herds, resulting in sepsis and sudden unexpected deaths [7,8]. In people who become infected with *E. rhusiopathiae* through direct cutaneous contact with animals, their carcasses (e.g., during necropsy), animal products, or feces, the disease most often presents in a mild skin form known as erysipeloid [5,9].

In Poland, *E. rhusiopathiae* infections are a significant problem in waterfowl breeding [10]. The pathogen causes erysipelas mainly in geese, and less often in ducks. These birds are bred outdoors, which increases their exposure to bacteria found in the environment (soil and water), including *E. rhusiopathiae*. The disease takes the form of sepsis and progresses rapidly, leading to death, and thus may result in substantial economic losses. Flock mortality ranges from 1% to even 50% and depends on how quickly appropriate treatment is implemented [10,11]. As standard erysipelas therapy involves administering antibiotics to birds, it is crucial to monitor the prevalence of resistant strains of *E. rhusiopathiae* and determine their resistance mechanisms, as well as the potential mechanisms of resistance gene transfer. This information is necessary for developing methods of controlling erysipelas in farm animals. Currently, available data on the antibiotic resistance, serotypes, and genotypic features of *E. rhusiopathiae* strains causing septicemia in waterfowl are very scarce. The only report on the antimicrobial susceptibility of *E. rhusiopathiae* isolates from domestic geese in Poland confirmed a high frequency of resistance to tetracyclines and fluoroquinolones, but no strain was multidrug-resistant (MDR) [12]. Studies on swine erysipelas in Poland and around the world have revealed the occurrence of *E. rhusiopathiae* isolates resistant not only to tetracyclines and fluoroquinolones but also to lincosamides, macrolides, and pleuromutilins [2,13]. In phenotypically resistant isolates, the presence of resistance genes such as *tetM*, *lnuB*, *lsaE*, *ant(6)-Ia*, *aph-A3*, *spw*, *ermT*, *ermA*-like, and *msrD*, as well as the occurrence of mutations in the *gyrA* and *parC* genes, has thus far been confirmed [2,13,14].

The main aim of this study was to analyze the whole-genome sequences (WGSs) of four strains of *E. rhusiopathiae* from domestic geese, including MDR strains, in order to detect resistance genes and associated mobile genetic elements, as well as to determine their sequence type (ST) and phylogenetic relationships. The comparative analyses included 363 *E. rhusiopathiae* strains and 13 strains of other *Erysipelothrix* species whose WGSs are publicly available.

## 2. Results and Discussion

### 2.1. Identification and Serotyping

The results of real-time PCR confirmed that the four isolates tested belong to the species *E. rhusiopathiae*. Moreover, all isolates contained *spaA* genes characteristic of the species, and their 16S rDNA sequences showed 100% homology to the 16S rDNA of the reference strain *E. rhusiopathiae* ATCC 19414 (GenBank acc. no. (GB): NR_040837.1).

Strains 1023 and 1012 represented serotype 2, while strains 267 and 584 belonged to serotype 5. These two serotypes, together with serotype 1b, are among the most common among *E. rhusiopathiae* strains isolated from geese and pigs in Poland [2,12].

### 2.2. Basic Genomic Analyses

**Figure A1:**
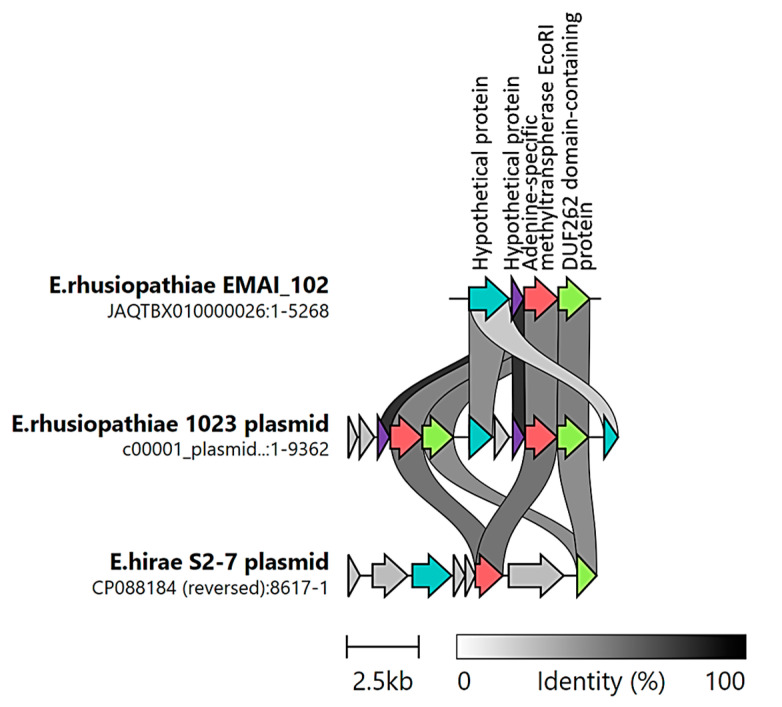
Clinker visualization of sequence homology between the plasmid of *E. rhusiopathiae* 1023 (products of all detected genes were designated as ‘hypothetical protein’) and genomic sequences of *E. rhusiopathiae* EMAI_102 (GB: GCA_029073445.1, query cover 85%, homology 88.93% [47]) and plasmid of *E. hirae* S2-7 (GB: CP088184.1, query cover 24%, homology 86.78%) [74]). Arrows with the same colors represent genes that are homologous to each other.

The assembled circular genomes of the four *E. rhusiopathiae* strains ranged from 1,826,410 bp to 1,905,426 bp, and the GC content was 36–37%. The number of genes ranged from 1720 to 1794, and each genome was estimated to contain 55 tRNAs with 3–7 rRNA loci. In strain 1023, a small plasmid of 9362 bp and GC content of 29% was detected (Table 1). The The Basic Local Alignment Search Tool (BLAST) analysis showed no homology between the plasmid identified and the *E. rhusiopathiae* plasmid sequences deposited in the GenBank database, i.e., the pAP1 plasmid with a length of 1987 bp (GB: NC_002148) and the pER29 plasmid with a length of 3749 bp (GB: KM576795.1). However, there was some homology between the plasmid of strain 1023 and the genomic sequences of *E. rhusiopathiae* strains EMAI_102, EMAI_40, and EMAI_82 from pigs in Australia, as well as the sequence of the *Enterococcus hirae* S2-7 plasmid (Figure A1). The plasmid identified did not contain resistance genes, and its function remains unknown (the products of all detected genes were designated as ‘hypothetical protein’).

Our results coincide with other reports and the data available in the GenBank database, according to which the size of the genomes of *E. rhusiopathiae* strains ranges from 1.6 to 1.9 Mb, and the GC content ranges from 36% to 38% (Table S1). The number of tRNAs ranges from 53 to 57 [15,16,17]. The occurrence of plasmids in *E. rhusiopathiae* strains ranging from 1.4 to 86 kb in size and of unknown function was originally described by Noguchi et al. [18]. Similar results were obtained by Pomerantsev et al. [19], who reported no correlation between the presence of plasmids (1.95, 2.2, 3.3, and 84 kb) and the drug susceptibility of *E. rhusiopathiae* strains. Xu et al. [20], however, demonstrated a small plasmid pER29 (3749 bp) in *E. rhusiopathiae* carrying the *ermT* gene determining macrolide resistance.

### 2.3. Antibiotic Susceptibility and Resistance Gene Profiles

The *E. rhusiopathiae* isolates differed in their antibiotic resistance profiles. Strains 1023 and 267 were found to be resistant to multiple antimicrobials, including erythromycin, tylosin, clindamycin, lincomycin, enrofloxacin, and tetracycline, and strain 1023 was additionally resistant to tiamulin; in addition, the MICs of streptomycin and spectinomycin were very high (>512 ug/mL). Strain 1012 showed resistance to two antibiotics, tylosin and tetracycline, and strain 584 was susceptible to all the antimicrobials used. All strains had very high MIC values for gentamicin (≥512 µg/mL), which is consistent with the findings of other authors and indicates intrinsic resistance to these antibiotics in *E. rhusiopathiae* [2,7,21] (Table 1 and Table S2).

The presence of the resistance genes detected in the four strains was correlated with their phenotypic resistance profile. The *tetM* gene coding for ribosome protection protein TetM was identified in the three tetracycline-resistant *E. rhusiopathiae* strains, i.e., 1023, 267, and 1012. The genome of MDR strain 1023 also contained the *erm47* gene (coding for methyltransferase) for macrolide, lincosamide, and streptogramin B (MLSB) resistance; the *lnuB* gene (lincosamide nucleotidyltransferase) for lincosamide resistance; the lsaE gene (ABC transporter) for tiamulin resistance; the *ant(6)-Ia* gene (aminoglycoside nucleotidyltransferase, *aadE*) for streptomycin resistance; and the spectinomycin resistance gene *spw* (aminoglycoside nucleotidyltransferase of ANT(9) family). Strain 267, in addition to the *tetM* gene, also contained the *ermB* gene responsible for bacterial resistance to macrolides and lincosamides. No resistance gene was detected in strain 584 (Table 1 and Table S1). In the two strains resistant to enrofloxacin (MIC ≥ 2), i.e., 1023 and 267, there was a mutation in the *gyrA* gene at position 257 (ACA→ATA or ACA→AAA), resulting in a Thr86→Ile or Thr86→Lys86 substitution (Table 1). The same mutations were previously noted in enrofloxacin-resistant strains of *E. rhusiopathiae* from pigs [2].

Among the 363 *E. rhusiopathiae* strains whose genomic sequences were obtained from the GenBank database, resistance genes were detected in 61 strains (16.8%), with the *tetM* gene detected most frequently (58/363, 16%). Only two strains (2/13) representing other *Erysipelothrix* species contained resistance genes, i.e., *tetM* and *tetT*. From 1.1% (*n* = 4) to 3.6% (*n* = 13) of *E. rhusiopathiae* strains contained the *lnuB*, *lsaE*, *ant(6)-Ia*, *spw*, *aph(3′)-III* and *lnuD-like* genes (Table S1 and Table 2). The last of these, found in four strains from pigs in China and Belgium, had 79% homology to the reference *lnu(D)* sequence of *Streptococcus uberis* (GB: EF452177.1) [22]. The remaining resistance genes, i.e., *bleO*, *aadD*, *aad9*, *spc*, *str*, *ermG*, *ermT*, *mefA*, *msrD*, and *tetT*, were identified in single genomes (Table 2).

One genome, that of strain *E. rhusiopathiae* 6026 from swine in Canada (1998), had an unusual genotypic resistance profile: *bleO-aadD-ant(9)-Ia-spc-lnuB*,*-lsaE-tetM* (Table S1). The *erm47* and *lnuB* genes identified in strains 1023 and 267, respectively, were not detected in any publicly available genomes (Table 2 and Table S1).

The occurrence of macrolide-resistant and MDR *E. rhusiopathiae* strains demonstrated in this study has rarely been observed [2,7,12,13,14,23,24]. Bobrek and Gaweł [12] reported no MDR or macrolide-resistant strains among 47 *E. rhusiopathiae* isolates from domestic geese in Poland, although several strains showed intermediate susceptibility to erythromycin. A few other studies confirmed the common susceptibility of *E. rhusiopathie* strains to macrolides [2,7,23]. In contrast, researchers from China [13] classified as many as 53% of isolates from pigs as resistant to macrolides. The *ermT* and *ermA-like* genes were detected in most of these strains (the *ermA-like* gene sequence was not publicly available and therefore could not be included in our study) [13]. An even higher percentage of *E. rhusiopathiae* strains resistant to macrolides (76.7%) was reported by Hess et al. [24] in Austria, but the genetic basis of this resistance was not investigated.

The *erm47* gene detected in *E. rhusiopathiae* isolate 1023 was originally described in 2016 in the clinical isolate *Helcococcus kunzii* UCN99 from a human diabetic foot ulcer in France (reference GB sequence: NG_054944.1). The protein encoded by the *erm47* gene shares 44–48% amino acid identity with known Erm methylases and determines constitutive resistance to MLSB [25]. The *erm47* sequence of strain 1023 is 99.6% homologous to the *erm47* of *Helcococcus kunzii* UCN99 (differing in 3/741 nt) as well as to the sequence of another *erm47*-positive strain found in the GenBank database, *Peptoniphilus* sp. ING2-D1G (GB: LM997412. 1) [26]. The location of the *erm47* gene in strain UCN99 on the 81 kb genomic island is consistent with the results of our research. The *ermB* gene detected in strain *E. rhusiopathiae* 267 encodes ribosomal methylase, which dimethylates a single adenine in 23S rRNA, leading to MLSB resistance. The *ermB* determinant is commonly found in Gram-positive bacteria (e.g., *Streptococcus* sp., *Staphylococcus* sp., *Enterococcus* sp., *Clostridium* sp., and *Lactobacillaceae*) and less frequently in Gram-negative bacteria (e.g., *Campylobacter* sp.) [27,28,29,30]. However, it has not yet been confirmed in *E. rhusiopathiae* strains. The remaining resistance genes detected in the strains tested, i.e., *tetM*, *lnuB*, *lsaE*, *ant(6)-Ia*, and *spw*, were previously confirmed in *E. rhusiopathiae* strains from pigs in Poland [2] and in China [13,14].

### 2.4. Identification of ICEs

**Figure 1:**
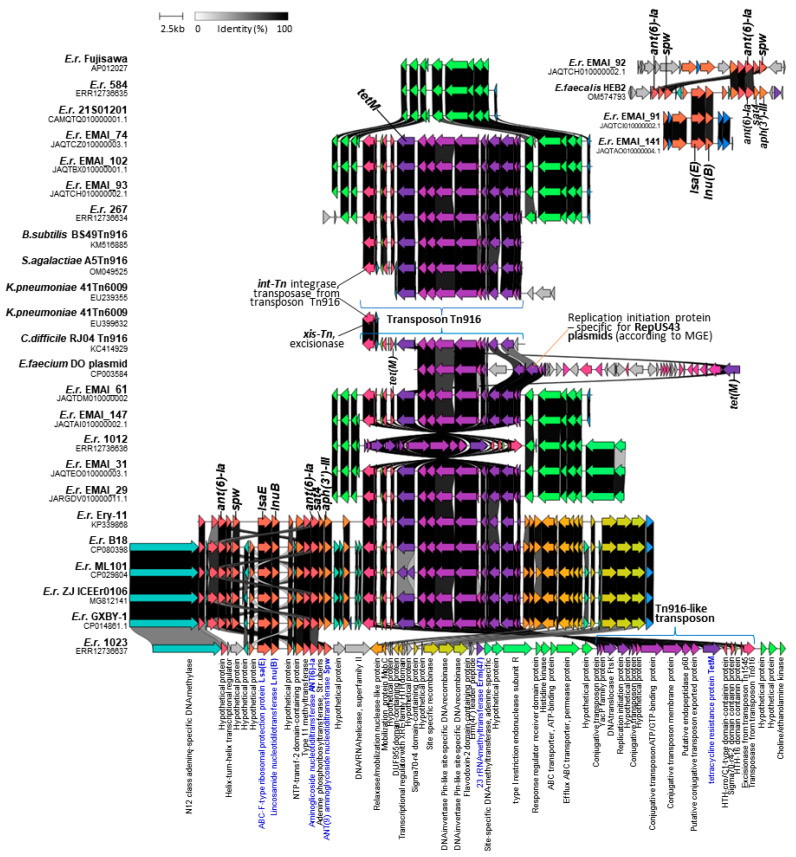
Clinker visualization of homology between genome sections of *E. rhusiopathiae* strains containing a Tn*916*-like transposon and resistance genes. The analysis also included the sequences of the repUS43 replicon of the *E. faecium* DO strain (GB: CP003584), fragments of the Tn6009 transposon of *K. pneumoniae* 41 (GB: EU239355 and EU239355), and Tn*916* transposon of *B. subtilis* BS49 (GB: KM516885.1), *S. agalactiae* A5 (GB: OM049525), and *C. difficile* RJ04 (GB: KC414929.1). MGE—tool for the detection of mobile genetic elements (https://cge.food.dtu.dk/services/MobileElementFinder/, accessed on 30 December 2023). Arrows represent genes; the arrow’s colors represent the gene clusters identified by Clinker; the grey arrows indicate an identity below 30%; homology between genes is represented by a gray gradient (%, the scale at the top of the figure); blue inscriptions concern genes that determine bacterial resistance to antimicrobias.

Analysis using the MobileElementFinder (MGE) tool v1.0.3 showed the presence of a Tn6009 transposon and the repUS43 plasmid in three (1023, 267, and 1012) of the four *E. rhusiopathiae* strains tested, as well as the colocalization of sequences specific for these mobile genetic elements with the *tetM* gene. The presence of a Tn6009 (*n* = 59) or Tn925 (*n* = 1) transposon and replicon repUS43-specific sequences was also recorded in all the *tetM*-positive *Erysipelothrix* spp. genomes (59/376) derived from the GenBank database. More detailed analyses showed that the *tetM* gene in *E. rhusiopathiae* strains is located within an ~18,000 bp DNA segment that is highly homologous (97.7–99.8%) to the Tn*916* transposon of *Enterococcus faecalis* DS16 (GB: U09422.1) [31], *Bacillus subtilis* BS49 (GB: KM516885.1) [32], *Clostridium difficile* RJ04 (GB: KC414929.1) [33], and *Streptococcus agalactiae* A5 (GB: OM049525) [34] and to the Tn6009 transposon fragment of *Klebsiella pneumoniae* 41 (GB: EU239355 and EU399632.1) [35] (Figure 1).

**Figure A2:**
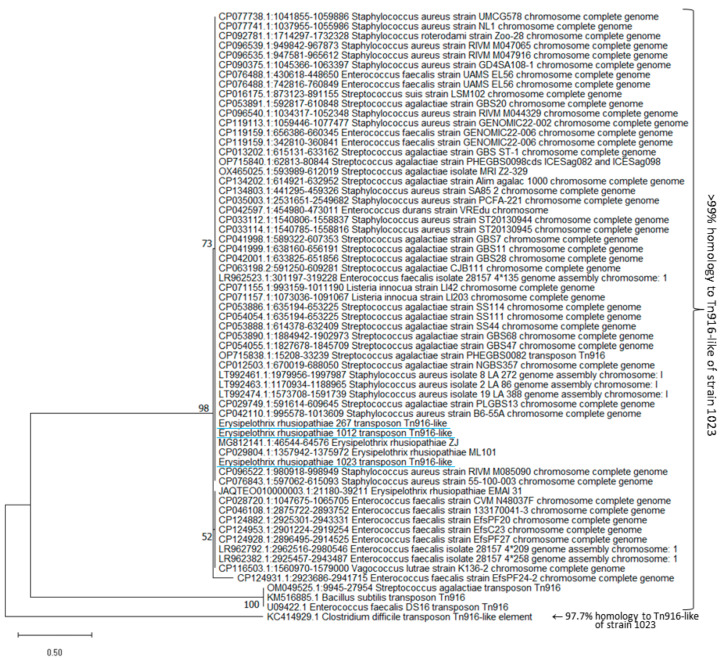
Dendrogram based on BLAST analysis showing the genetic similarity of the transposon sequences identified in *E. rhusiopathie* strains 1023, 1012, and 267 (underlined in blue) with sequences deposited in GenBank.

The absence of the *tetM* gene in *E. rhusiopathiae* strains corresponded to the absence of the entire transposon. BLAST analysis showed that sequences homologous to transposons (~18,000 bp) found in *E. rhusiopathiae* strains 1023, 1012, and 267 are also present in many strains of *Staphylococcus aureus*, *Enterococcus faecalis*, *Streptococcus agalactiae*, *Streptococcus suis*, and *Listeria inocula* (Figure A2). Taking into account the high homology of the sequences found in the genomes of *E. rhusiopathie* strains to the Tn*916* transposons and the fact that the MGE tool assigns the transposon to the Tn6009 type solely on the basis of a short sequence (an 1889 bp section of the Tn6009 transposon of *K. pneumoniae* 41, GB: EU399632.1), which is 99.9% homologous to the sequence present within the transposons of the Tn*916* family (GB: U09422.1; KM516885.1, KC414929.1, and OM049525) (Figure 1), the transposons detected in *E. rhusiopathiae* strains were ultimately designated as Tn916-like. Moreover, the Tn6009 transposon is much larger than those detected in *E. rhusiopathiae* genomes; it is ~23 kb long [36] and contains the Tn*916* element linked to the *S. aureus* mer operon carrying genes encoding resistance to mercury [35].

As shown in Figure 1, the Tn*916*-like conjugative transposons in *E. rhusiopathiae* strains are integrated at a specific site on the bacterial chromosome. This is consistent with previous reports showing that the Tn*916* transposon integrase binds to bacterial sequences known to be target sites for Tn*916* insertion. These are regions usually rich in AT, and they are used with varying frequency in Gram-positive bacteria [37,38].

The analysis using the MGE tool v1.0.3 showed that the Tn*916*-like transposons found in *E. rhusiopathiae* strains contain sequences specific to the repUS43 replicon corresponding to the gene encoding the ‘replication initiation protein’ in plasmid 1 of the *Enterococcus faecium* DO strain (also known as strain TX16) (GB: CP003584: 24,026–25,231). It should be noted that this sequence is only 1206 bp long, and the entire plasmid 1 is 36,262 bp long [39] (Figure 1). In other bacteria, the repUS43 plasmid-specific sequence may be located within Tn*916*-type transposons inserted either into the chromosome (e.g., *Streptococcus gallolyticus* ATCC 43143, GB: AP012053.1) [40] or into the plasmid (e.g., plasmid pCTN1046 *Ligilactobacillus salivarius*, GB: CP007650.1) [41]. Given the above, the reliability of the MGE results confirming the presence of repUS43 plasmids in all Tn*916*-positive strains can be considered highly questionable.

**Figure 2:**
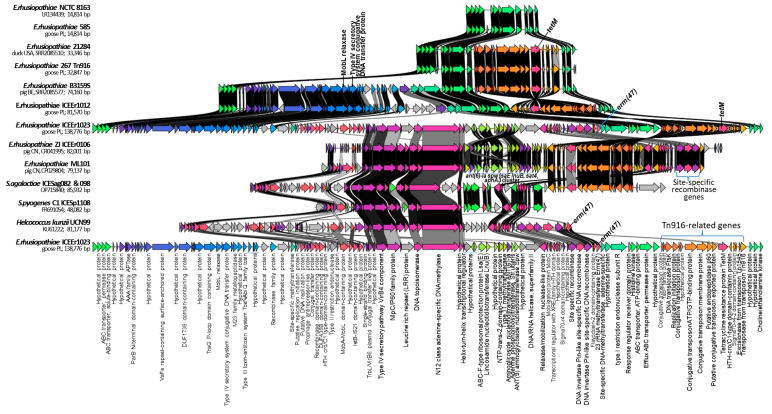
Genetic context of integrative and conjugative elements (ICEs) of *E. rhusiopathiae* strains (267, 1012, and 1023) compared with ICEs found in other *E. rhusiopathiae* strains and other Gram-positive bacteria. The arrow’s colors represent the gene clusters identified by Clinker. Black background indicates identity between gene sequences.

**Figure A3:**
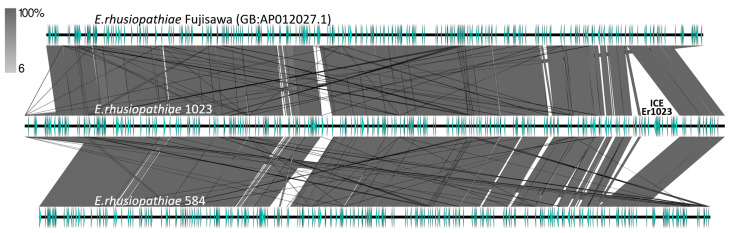
Easyfing alignment of genome sequences of strain 1023 containing ICE Er1023 (~130 kb) and strains *E. rhusiopathiae* 584 and Fujisawa (GB: AP012027.1 [16]). Green arrows represent the locations of coding sequences and shaded lines reflect the degree of homology between genomes.

**Figure A4:**
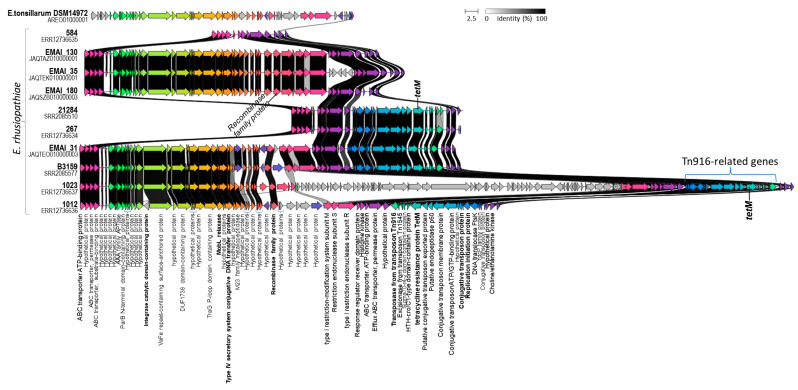
Comparison of ICE sequences identified in the tested strains of *E. rhusiopathiae* from geese (1023, 1012, and 267) with ICEs found in genomes of other *E. rhusiopathiae* strains and one *E. tonsillarum* strain. The arrow’s colors represent the gene clusters identified by Clinker.

A comparative analysis of the sequences of the entire genomes of the four *E. rhusiopathiae* strains tested in this study showed the presence of large integrative and conjugative-like elements in strains 1012 and 1023, with lengths of 74 kb (ICEEr1012) and 130 kb (ICEEr1023), respectively (Figure 2 and Figure A3). The GC content in these elements was slightly lower than in the bacterial chromosome (36%) and amounted to 34%. The ICEs showed homology to each other, but ICEEr1023 contained an additional segment of ~66 kb, within which the *lsaE-lnuB-ant(6)-Ia-spw* cluster and *erm47* gene resistance gene were located. Both large ICEs contained a Tn*916*-like transposon (~18 kb) and transfer genes characteristic of conjugative plasmids, i.e., genes encoding recombinases, relaxases (MobL and MobL-MobA domain-containing protein), and the IV secretion system (T4SS). Both ICEs were located at a specific site of the bacterial chromosome, which indicates the involvement of site-specific recombinases in their integration. Indeed, genes of three site-specific recombinases were identified within ICEEr1023, and both ICEs contained the integrase and excisionase genes of the Tn*916* transposon (Figure 2 and Figure A4).

According to the generally accepted definition, ICEs are integrated into the host chromosomes, but they can excise from them, form circular structures, and transfer (via conjugation) to neighboring cells [42]. Most genes related to the ICE life cycle are not expressed when the ICE is integrated into the chromosome. However, under certain conditions, or perhaps spontaneously, the expression of the ICE genes needed for excision and conjugation is induced [42]. Relaxases, also called Mob (mobilization) proteins, initiate bacterial conjugation through a site- and strand-specific nick in the oriT region of the conjugation element. This generates a single-stranded DNA molecule that is transferred from the donor to the recipient cell via the T4SS multicomponent protein pore. MobL relaxases are found mainly in Firmicutes and are believed to play a key role in horizontal gene transfer in these bacteria [42,43]. ICEs integrate into and excise from DNA using an ICE-encoded recombinase. This enzyme is often homologous to phage integrases, and, like temperate phages, many ICEs insert at a specific attachment site in the bacterial chromosome (*attB*). For many ICEs, *attB* is found in a tRNA gene [42]. However, this location was not confirmed for ICEEr1023 and ICEEr1012. ICEs, in addition to genes related to their life cycle, typically also contain cargo genes, including virulence-related genes and antimicrobial resistance genes that confer the phenotype to host cells [42]. The structure of the ICEs detected in this study in *E. rhusiopathiae* strains is fully consistent with this information. It should be noted, however, that despite the presence of genes related to integration, excision, and conjugation within ICEEr1023 and ICEEr1012, we did not confirm their capacity for excision and conjugal transfers. Therefore, these issues require further in-depth research.

ICEEr1023 was homologous to ICEEr0106 of *E. rhusiopathiae* strain ZJ (GB: MG812141) and ICEs found in strains ML101 (GB: CP029804) (Figure 2), GXBY-1 (GB: CP014861) [17] and B18 (GB: CP080398.1) [13] (Figure 1) from pigs in China (>99%, query cover 40%). As with ICEEr1023, these ICEs contained the Tn*916*-like transposon with the *tetM* gene, three site-specific recombinase genes, and the *lsaE-lnuB-aadE-spw* cluster, which in these strains was expanded by an additional copy of the *aadE* gene, and the *sat4* and *aph(3’)-III* genes [14]. It should be noted, however, that ICEEr1023 (130 kb) was longer than ICEEr0106 (79 kb) and the ICE of the strain ML101 (79 kb) by approximately 51 kb and contained the *erm47* gene. The initial fragment of ICEEr1023, 38 kb long, which contained the genes encoding MobL relaxase and the type IV secretory system, showed no homology to the ICEs of the above-mentioned strains from China (Figure 2). Transposon Tn*916* (originally defined as Tn*5251*) and the above-mentioned resistance gene cluster were also previously described in the *E. rhusiopathiae* Ery-11 (GB: KP339868) strain from pigs in China [44] (Figure 1). A similar cluster was detected in several other genomes of *E. rhusiopathiae* examined in the present study, but it contained fewer resistance genes, i.e., 2–3, with the *lnuB* gene always occurring together with the *lsaE* gene, and the *ant(6)-Ia* gene together with the *spw* gene (Figure 1 and Table S1). The *aadE–spw–lsa(E)–lnu(B)* cluster is also found in other Gram-positive bacteria, i.e., enterococci, streptococci, and staphylococci, and may be located on a plasmid or the bacterial chromosome within the integrative conjugative element [45]. ICEEr1023 also showed homology to ICE sequences found in other Gram-positive bacteria, i.e., *S. agalactiae* PHEGBS0098 (GB: OP715840), *S. pyogenes* C1 (ICESp1108 and FR691054) and *Helcococcus kunzii* UCN99 (GB: KU61222) (Figure 2). The last of these, like ICEEr1023, contained the *erm47* gene.

BLAST analysis showed that sequences highly homologous to ICEEr1012 are also present in the genomes of *E. rhusiopathiae* EMAI_31 (from a pig, Australia), B3159S (from a pig, Belgium) and 21284 (from a duck, USA). Moreover, in several other *E. rhusiopathiae* strains (EMAI: 35, 130, 131, 133, 134, 135, 159, 171, 172, and 180) and in the *E. tonsillarum* DSM 14972 strain, regions homologous to ICEEr1012 but lacking the gene set of the Tn*916*-like transposon were detected (Figure A4). Such ICEs were 49–54 kb long, did not contain resistance genes, and were located at a specific site of the chromosome (Figure A4 and Figure 2).

### 2.5. Detection of Phage DNA and Its Possible Involvement in the Transduction of Resistance

Prophage DNA was detected in genomes of all the tested *E. rhusiopathiae* strains (1023, 1012, 267, and 584) but was considered intact only in strain 548 (score 130). *Erysipelothrix* phage SE-1 (BG: NC_029078.1) was the most common prophage. Detailed information on the prophage DNA detected in the genomes of the *E. rhusiopathiae* strains, including length, GC content, and number of coding sequences, is shown in Table A1.

**Figure 3:**
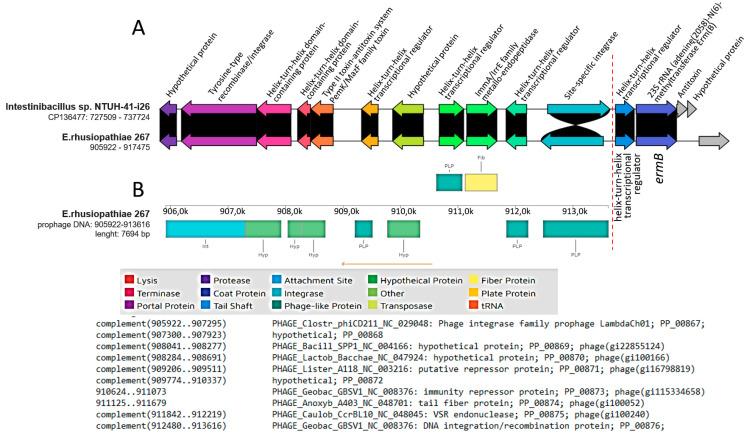
Location of prophage DNA and the *ermB* gene in the genome of *E. rhusiopathiae* 267” (**A**) clinker visualization of BLAST comparative analysis of the sequence of *E. rhusiopathiae* 267 containing prophage DNA and the homologous sequence of *Intestinibacillus* sp. NTUH-41-i26; (**B**) results of the analysis of phage DNA of strain 267 using PHASTER (https://phaster.ca, accessed on 12 February 2024). Red dashed line separates phage DNA from bacterial DNA. Black background indicates identity between gene sequences.

Prophages did not harbor resistance genes; however, in strain 267, the phage DNA was located next to the *ermB* gene sequence (Figure 3). Hence, the phage seems very likely to be involved in the transmission of this gene. A sequence homologous to the section of the genome of strain 267 corresponding to phage DNA and the *ermB* gene was also detected in *Intestinibacillus* sp. strain NTUH-41-i26 (CP136477.1) (Figure 3).

The *ermB* gene is usually located on mobile genetic elements such as transposons or other ICEs [28], and the involvement of bacteriophages in its dissemination is poorly understood. Wang et al. [46], however, showed that the *ermB* gene was present in 100% of phage DNA samples from pig farms in China and was the most abundant gene in the population of bacteria inhabiting these environments. These data indicating the possibility of horizontal transfer of the *ermB* gene by transduction are consistent with our results. Previously, the presence of prophage DNA was demonstrated in the genome of *E. rhusiopathiae* WH13013 (the phage type was not determined) [15] and *E. rhusiopathiae* ZJ from pigs from China [14]. In the latter case, the prophage DNA was 86 kb in size and contained genes determining bacterial resistance to macrolides, i.e., *mef(A)* and *msr(D)*. Moreover, in vitro experiments showed that this phage, designated Φ1605, can, with the participation of mitomycin C, infect other strains of *E. rhusiopathiae* and transmit the above-mentioned resistance genes [14].

### 2.6. MLST Results

MLST analysis identified the STs for strains 1023 and 584 as ST 4 and ST 32, respectively. Strains 1012 and 267 were not assigned an ST in the current scheme, with a novel allele for *recA* identified in strain 267 and a unique combination of alleles observed in strain 1012. Consequently, novel STs were assigned: ST 243 for strain 267 and ST 242 for strain 1012. Of the 363 other *E. rhusiopathiae* strains whose WGSs were included in the comparative analyses, the ST had already been determined for 178 [47]. For 8 of the remaining 184 strains, the ST could not be clearly determined due to incomplete sequencing (Table S1). ST 4, detected in strain 1023, has also been confirmed in several strains from pigs, including strain 6106 from Canada (GB: SRR2085525), EMAI_89 from Australia (GB: GCA_029073745.1), and four strains from Europe (isolates 174, 175, and 17MIK0642311 from Italy and isolate 16BKT31008 from Denmark). ST 32, represented by strain 584, has also been identified in a strain called swine20 (GB: ERR3678840) from a pig in the UK [48] (Table S1). In one *E. rhusiopathiae* strain, i.e., DISL19 from a dolphin in the USA, a new allele combination was recorded and designated as type 244.

**Figure 4:**
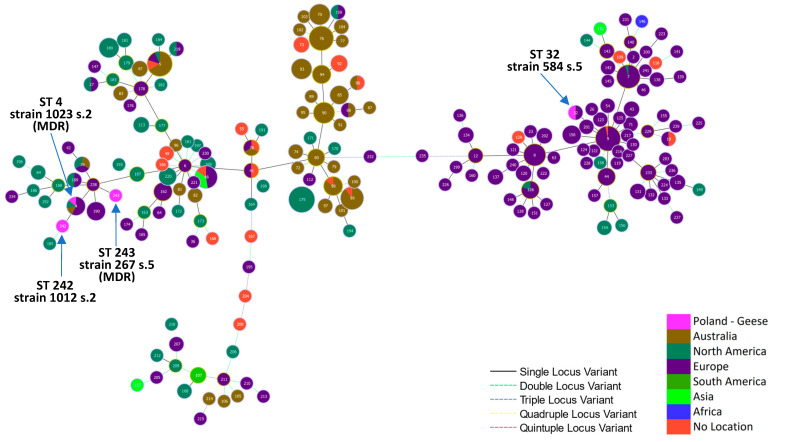
Minimum spanning tree of an *E. rhusiopathiae* MLST scheme created from the sequence of genes *pta*, *galK*, *purA*, *ldhA*, *recA*, *gpsA*, and *prsA*. Two major single-locus variants (SLVs) consisting of a European group of isolates and a second SLV of isolates from Australia and the Americas; s.—serotype.

Strain 584, representing ST 32, was located in a large single-locus variant (SLV) group, consisting mainly of isolates originating in Europe, while the remaining three goose strains, representing ST 4, 242, and 243, were located in a group containing mainly isolates from North America and Europe (Figure 4).

The STs of the goose strains were not consistent with the STs of 16 *E. rhusiopathiae* strains from poultry (1 strain from duck in the USA and 15 strains from an unspecified poultry species in Belgium and Canada) nor with the STs of several strains from wild birds (*n* = 8) included in the comparative analyses. Strains from poultry represented thirteen different STs, with ST5 recorded in three of them (Table S1, Figure 4). The same sequence type, alongside ST 76 and ST 99, predominated among *E. rhusiopathiae* strains from pigs in Australia [47].

### 2.7. Phylogenetic Inference

**Figure 5:**
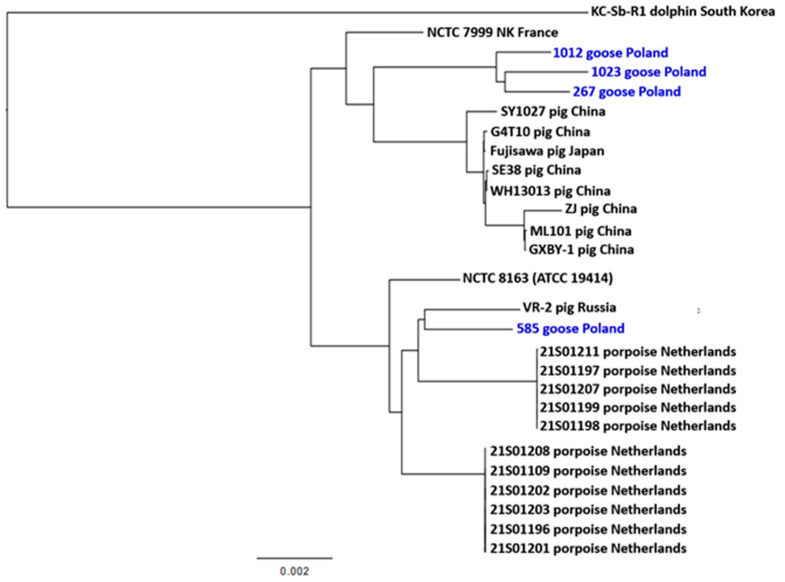
Phylogenetic tree of a 1,075,094 bp core-gene super-alignment of *E. rhusiopathiae* strains from geese, Poland (*n* = 4), compared with 23 genomes of *E. rhusiopathiae* strains whose sequences were taken from the GenBank database (the accession numbers of these sequences are listed in Table S1). The blue font refers to the strains tested in this work.

The phylogenetic inference of 27 *E. rhusiopathiae* strains was performed on a 1210-core-gene super-alignment identified using Roary v3.13.0. Strains 1023, 1012, and 267 formed a separate clade, differing in ~226–1070 SNPs, while strain 584 was located in a different part of the dendrogram and showed high relatedness to strain VR-2 isolated from a pig in Russia [49] (Figure 5). The results of the analysis indicate that the tested *E. rhusiopathiae* strains belong to two clonal lineages. Strains from the Netherlands as well as strains from China and Japan formed separate clades, which indicates a certain relationship between geolocation and the structure of the core genome in *E. rhusiopathiae*. Similar results of phylogenetic analysis were presented by Yang et al. [15], who showed close relatedness between strains originating in pigs in China (WH13013, ZJ, ML101, GXBY-1, and Sy1027) and Japan (Fujisawa) and a large phylogenetic distance between these strains and the KC-Sb-Ra strain from South Korea, as well as the reference strain *E. rhusiopathiae* NCTC 8168. The phylogenetic distinctness of strains from porpoises in the Netherlands compared to the strains isolated from pigs in Great Britain was noted in the work of IJsseldijk et al. [4].

## 3. Materials and Methods

### 3.1. Isolation, Identification, and Phenotypic Characterization of E. rhusiopathiae Strains

The four strains of *E. rhusiopathiae* included in this study, i.e., 1023, 1012, 267, and 584, were isolated from the internal organs of dead domestic geese from various Polish farms. Bacteria were cultured on Columbia medium supplemented with 5% sheep blood at 37 °C and 5% CO_2_, and their taxonomic identity was confirmed based on the results of a real-time PCR test (EXOone *Erysipelothrix rhusiopathiae*, Exopol, Spain) and the identification of the *spaA* gene [2]. In addition, 16S rRNA gene sequence analysis was performed (based on analysis of WGSs).

These 4 strains were selected from a pool of 60 *E. rhusiopathiae* isolates from waterfowl based on antimicrobial susceptibility test (AST) results (unpublished data). Strains with different antimicrobial susceptibility profiles were intentionally selected, i.e., two MDR strains (1023 and 267, resistant to three or more classes of antimicrobials), one strain resistant to two antimicrobials (1012), and one strain susceptible to all antimicrobial agents (584). The AST was performed using the broth microdilution method as described previously [2]. Isolates were tested against penicillin, ampicillin, ceftiofur, tetracycline, erythromycin, tylosin, clindamycin, lincomycin, tiamulin, enrofloxacin, streptomycin, spectinomycin and gentamicin. The categorization of *E. rhusiopathiae* strains as susceptible and resistant was based on the Clinical & Laboratory Standards Institute (CLSI) guidelines [50], and in the case of antimicrobials not included in this guide (lincomycin and tiamulin), the recommendations of Dec et al. [2] were used. The categorization did not include streptomycin and spectinomycin due to the lack of available guidelines.

The serotyping of the four *E. rhusiopathiae* strains was performed according to a previously developed multiplex PCR protocol (for the detection of serotypes 1a, 1b, 2, and 5) [51].

### 3.2. Whole-Genome Sequencing

DNA was isolated using a DNeasy UltraClean microbial kit (Qiagen, Venlo, The Netherlands). Nanopore sequencing was performed according to protocol SQK-RBK110.96 with Flow Cell version R9.4.1 on a MinION device (FLO-MIN106D; Oxford Nanopore, Oxford, UK), using the super-accurate base-calling method in MinKNOW v22.12.7. Reads were trimmed and downsampled to 200× coverage using filtlong (https://github.com/rrwick/Filtlong, accessed on 6 January 2024) and assembled into circular contigs using Flye v2.9.1 [52]. Genomes were polished using Medaka v.1.1.0 (https://github.com/nanoporetech/medaka, accessed on 6 January 2024) and Homopolish (https://github.com/ythuang0522/homopolish?tab=readme-ov-file, accessed on 6 January 2024) [53] and annotated using Prokka v1.14.5 [54].

### 3.3. WGSs Used in Comparative Analysis and Determination of Homology between DNA Sequences

The comparative analysis included a total of 363 WGSs of *E. rhusiopathiae* strains and 13 WGSs of other *Erysipelothrix* species from the GenBank database (Table S1). The sequences of 148 of these genomes were downloaded from GenBank’s Sequence Read Archive (SRA) and assembled using SPAdes genome assembler v3.14.1 [55].

Alignment between the genomic sequences of the four *E. rhusiopathiae* strains and the reference strain *E. rhusiopathiae* Fujisawa was performed using Easyfig v.2.2.5 [56]. The comparative analysis of resistance genes, integrative conjugative elements, and plasmids detected in the genomes of the *E. rhusiopathiae* isolates was carried out using BLAST (https://blast.ncbi.nlm.nih.gov/Blast.cgi, accessed on 12 February 2024) and Cinker (https://cagecat.bioinformatics.nl, accessed on 9 February 2023) [57].

### 3.4. Multilocus Sequence Typing

The multilocus sequence typing (MLST) of *E. rhusiopathiae* strains was performed according to the scheme originally developed by Janßen et al. [58]. The analysis included seven housekeeping genes: *pta*, *galK*, *purA*, *ldhA*, *recA*, *gpsA*, and *prsA*. Genomes were screened against the MLST scheme updated by Webster et al. [47] using mlst v.2.19.0 (https://github.com/tseemann/mlst, accessed on 18 January 2024). A minimum spanning tree based on MLST allele numbers was computed in PHYLOViZ Online with the goeBURST full MST function, as previously performed by Webster et al. [47]. The analysis included a total of 367 *E. rhusiopathiae* strains (the 4 strains tested in this work and 363 strains whose genomic sequences are deposited in the GenBank database, Table S1), of which 178 had their sequence type previously determined by Webster et al. [47].

### 3.5. Detection of Resistance Genes, Mobile Genetic Elements, and Phage DNA

Resistance genes, plasmids, and integrative conjugative elements (ICEs) were identified in the genomes of the four *E. rhusiopathiae* strains using ABRicate software (https://github.com/tseemann/abricate, accessed on 2 February 2024) [59], Resfinder 4.1 [60], and MobileElementFinder (MGE v1.0.3) [61]. The *spw* gene was detected using the BLAST tool available on the National Center for Biotechnology Information (NCBI) web server (https://blast.ncbi.nlm.nih.gov/Blast.cgi, accessed on 12 February 2024). Synonymous mutations in the *gyrA* gene were determined by aligning amino acid sequences predicted using ORF Finder (https://www.ncbi.nlm.nih.gov/orffinder/, accessed on 17 February 2024).

Prophage DNA was detected in the genomes of the four *E. rhusiopathiae* strains using the PHAge Search Tool—Enhanced Release (PHASTER) (https://phaster.ca, accessed on 12 February 2024) [62].

### 3.6. Phylogenetic Inference

The phylogenetic analysis included the 4 tested *E. rhusiopathiae* strains from geese and 24 randomly selected *E. rhusiopathiae* strains whose genomic sequences were downloaded from the GenBank database (Table S1). Gene orthology and core-gene super-alignment were determined using Roary v3.13.0 [63] and visualized using GrapeTree (https://github.com/achtman-lab, accessed on 23 January 2024). Phylogenetic trees were constructed using Fasttree v 2.1.11 [64]. Trees were visualized using the Interactive Tree Of Life (iTOL) v5 [65].

## 4. Conclusions

This is the first report of the occurrence of macrolide-resistant and MDR strains of *E. rhusiopathiae* in waterfowl in Poland, as well as the only report presenting the WGSs of *E. rhusiopathiae* strains from geese. We were the first to demonstrate the presence of resistance genes *erm47* and *ermB*, ICEEr1023, and ICEEr1012, and prophage DNA related to the *ermB* gene in *E. rhusiopathiae* bacteria. We also showed that the *tetM* gene in *E. rhusiopathiae* is always located within the Tn*916*-like transposon. Novel STs, i.e., 242 and 243, were confirmed in two of the strains (1012 and 267). Our findings will be helpful in understanding the potential mechanisms of resistance gene transfer in *E. rhusiopathiae* and Gram-positive bacteria in general and highlight the need to investigate more clinical isolates of *E. rhusiopathiae* from waterfowl in order to determine their biodiversity and the spread of drug-resistant strains, including MDR strains. Phenotypic analyses and the detection of resistance genes by PCR can be very helpful in determining the antibiotic resistance of *E. rhusiopathiae*, but only WGS analyses provide detailed data on the colocalization of resistance genes and the occurrence of mobile genetic elements (ICEs and prophage DNA) containing resistance determinants. Data in this area not only increase knowledge about the characteristics of *E. rhusiopathiae* bacteria but are also needed to develop effective methods for controlling erysipelas in waterfowl. Further research is needed to confirm the capacity of ICEEr1023 and ICEEr1012 for excision/reintegration and conjugal transfer.

## Figures and Tables

**Table 1:** Results of antibiotic susceptibility testing and genomic analysis of *E. rhusiopathiae* isolates.

Isolate ID (Genome ID)	1023 (23S00 176-1)	267 (23S00171-1)	1012 (23S00175-1)	584 (23S00173-1)
Serotype	2	5	2	5
Source	Domestic goose	Domestic goose	Domestic goose	Domestic goose
Year of isolation	2021	2021	2020	2020
ENA Acc. No.	ERR12736637	ERR12736634	ERR12736636	ERR12736635
Genome size (bp)	1,905,426 ^a^; 9362 ^b^	1,889,255 ^a^	1,846,905 ^a^	1,826,410 ^a^
Contigs	2	1	1	1
Genes	1862	1835	1805	1790
Proteins (CDS)	1794	1767	1743	1720
tRNAs	55	55	55	55
GC content (%)	36 ^a^ 29 ^b^	37	36	37
ST (MLST)	4	243 (novel)	242 (novel)	32
Phenotypic resistance profile (MIC in μg/mL) ^c^	ERY (>32), TYL (2), LIN (>64), CLI (2), TIA (>64), ENR (>16), TET (64), …………………….. STR (>512), SPE (>512), GEN (>512)	ERY (>32), TYL (>32), LIN (>64), CLI (>16), ENR (8), TET (64), …………………… STR (128), SPE (32), GEN (512)	TYL (2), TET (32), ……………………. STR (64), SPE (128), GEN (>512)	………………….. STR (64), SPE (32), GEN (512)
Resistance genes	*erm47*, *lnuB*, *lsaE*, *ant(6)-Ia*, *spw*, *tetM*	*ermB*, *tetM*	*tetM*	none
Mutations in *gyrA* gene	Thr86→Ile86	Thr86→Lys86	Thr86	Thr86
ICE	Tn*916*-like, ICEEr1023	Tn*916*-like	Tn*916*-like, ICEEr1012	none
Plasmid	1	0	0	0

^a^ Bacterial chromosome, ^b^ plasmid; ^c^ MIC values below the dashed line refer to antimicrobials for which there are no cut-off points; ERY—erythromycin; TYL—tylosin; LIN—lincomycin; CLI—clindamycin; TIA—tiamulin, ENR—enrofloxacin; TET—tetracycline; STR—streptomycin; SPE—spectinomycin; GEN—gentamycin; NEO—neomycin; TR/S—trimethoprim/sulfamethoxazole; ENA—European Nucleotide Archive; ICE—integrative and conjugative element.

**Table 2:** Resistance genes detected in the genomes of *Erysipelothrix rhusiopathiae* and other *Erysipelothrix* species.

	*bleO*	*aadD*	*ant(9)-Ia* *(aad9)*	*spc*	*str*	*spw*	*ant(6)-Ia*	*aph(3′)-III*	*lnuB*	*lsaE*	*ermG*	*ermT*	*mefA*	*msrD*	*lnuD-like*	*erm47*	*ermB*	*tetT*	*tetM*
*E. rhusiopathiae* strains tested in this work *n* = 4	0	0	0	0	0	25% (1)	25% (1)	0	25% (1)	25% (1)	0	0	0	0	0	25% (1)	25% (1)	0	75% (3)
*E. rhusiopathiae* *n* = 363 *	0.3% (1)	0.3% (1)	0.5% (2)	0.3% (1)	0.5% (2)	3.3% (12)	3.3% (12)	1.9% (7)	3.6% (13)	3.6% (13)	0.3% (1)	0.3% (1)	0.3% (1)	0.3% (1)	1.1% (4)	0	0	0.3% (1)	16% (58)
*Erysipelothrix* sp. *n* = 13 *	0	0	0	0	0	0	0	0	0	0	0	0	0	0	0	0	0	7.7% (1)	7.7% (1)

* Genomes derived from the GenBank database (according to Table S1); the 13 strains identified as *Erysipelothrix* sp. represent species other than *E. rhusiopathiae*.

## Data Availability

The whole-genome sequences of the *E. rhusiopathiae* strains examined in this study (1023, 1012, 267, and 584) have been deposited in the European Nucleotide Archive under Project accession number PRJEB72737.

## Supplementary Materials

The following supporting information can be downloaded at https://www.mdpi.com/article/10.3390/ijms25094638/s1. References [66,67,68,69,70,71,72,73] are cited in the Supplementary Materials.

## Appendix A

**Table A1:** Results of genomic analysis of *E. rhusiopathiae* isolates, including the location of prophage DNA.

Strain ID	Prophage DNA Location	Length [kb]	Score *	Total Proteins	Most Common Phage	GC Content	Presence of Resistance Genes
267	352550…374967	22.4	50	26	PHAGE_Erysip_SE_1 NC_029078(6)	37.51%	No
	905922…913616	7.6	30	10	PHAGE_Geobac_GBSV1 NC_008376(2)	42.38%	*ermB* was found next to phage DNA (914 218…914 955; length 738 bp)
584	617896…660749	42.8	130	43	PHAGE_Erysip_SE_1 NC_029078(34)	35.13%	NA
	548403…566281	17.8	50	22	PHAGE_Erysip_SE_1 NC_029078(6)	37.63%	NA
1012	1614477…1637026	22.5	60	26	PHAGE_Erysip_SE_1 NC_029078(6)	37.45%	No
1023	709331…735594	26.2	20	21	PHAGE_Bacill_BCJA1c NC_006557(6)	34.68	No

* Score > 90—intact; score 70–90—questionable; score < 70—incomplete; NA—not applicable.

**Table A2:** The *recA* gene sequence (new allele) of strain 267.

CTCTTATTGATAAAGTCGTATTGAACACCTAAGTCGATTACTTCACCAATATAGCTAATTCCCTTACCGTAAATAATTTCAACTTGTGTTGCT CTAAAGGGCGGTGCCACTTTATTCTTTACGACTTTAATATTAGCTTTATTACCCACAATATCTGTACCTTGTTTTATTTGTTCACTACGACGA ATATCAAGTCGAACAGAAGAATAGAATTTTAAGGCACGTCCTCCCGGTGTTGTTTCAGGATTACCAAACATAATACCTACTTTTTCTCGA AGTTGATTAATAAAAATTGCAGTACACTCGCCTCGGTTCATTCCACCGGATAATTTACGCATTGCTTTAGACATCATCCGAGCTTGTAAAC CAACTTGAGCATCGCCCATTTCCCCATCAAGTTCCGCTTGTGGAACAAGTGCAGCAACACTGTCCACCACAATCAAGTCAACCGCACCAC TTCGTACCAAAACATCCACAATTTCCAATCCTTGTTCACCGCTATCGGGTTGTGAAAGAATTAAATCATCAATGTTAACGCCTAAGTTTTGA GCATAGATAGGATCAATCGCGTTCTCAGCATCGATAAATGCAGCTTTTCCACCCGCTTTTTGCACTTCTGCAATTGCATGTAATGCGAGCG
